# Supplementary material for: Genome-Wide Identification of the PAL Gene Family in Camellia nitidissima and Functional Characterization of CnPAL1 Gene by In Vitro Expression
Source: Genes (Basel). 2025 Oct 23;16(11):1251. doi: 10.3390/genes16111251 (PMC12652097; doi:10.3390/genes16111251)
Supplement: Supplementary file 1 [file genes-16-01251-s001.zip › Table S1 Primers used for qRT-PCR and cloning of CnPAL1 gene.pdf]

Table S1 Primers used for qRT-PCR and cloning of *CnPAL1* gene

Name of the primer	Sequence of the primer(5' to 3')	Application
CnPAL1-F	CCAACATCCTTGCCATCCTCT	Fluorescent quantitative
CnPAL1-R	AGCTTCTTTGACATAGGCGCT	
CnPAL2-F	TCAGAGCGTTTGAGGAGGAAC	
CnPAL2-R	GCCTTCTCTCCGGTCAGAAAA	
CnPAL3-F	GGTGACAACTGGATTTGGTGC	
CnPAL3-R	TTGCTGCTGACTGAGGAAGAG	
CnPAL4-F	GGATCAACACCCTCCTCCAAG	
CnPAL4-R	AGCCCGGCAATGTAAGATAGG	
CnPAL5-F	ATTAGCCGCGGAGTCAATGAA	
CnPAL5-R	CCACTCACTGCTTGCTTGAAC	
CnPAL6-F	ATTAGCCGCGGAGTCAATGAA	
CnPAL6-R	CCACTCACTGCTTGCTTGAAC	
18S rRNA-F	GACTCAACACGGGGAAACTTACC	qRT-PCR reference gene
18S rRNA-R	CAGACAAATCGCTCCACCAAC	
CnPAL1-F	ATGGAAATCACTAATGGTCACT	Clone
CnPAL1-R	GCAGATTGGAAGAGGAGCTC	
