# Supplementary figures and images for: Genome-Wide Identification of the PAL Gene Family in Camellia nitidissima and Functional Characterization of CnPAL1 Gene by In Vitro Expression
Source: Genes (Basel). 2025 Oct 23;16(11):1251. doi: 10.3390/genes16111251 (PMC12652097; doi:10.3390/genes16111251)

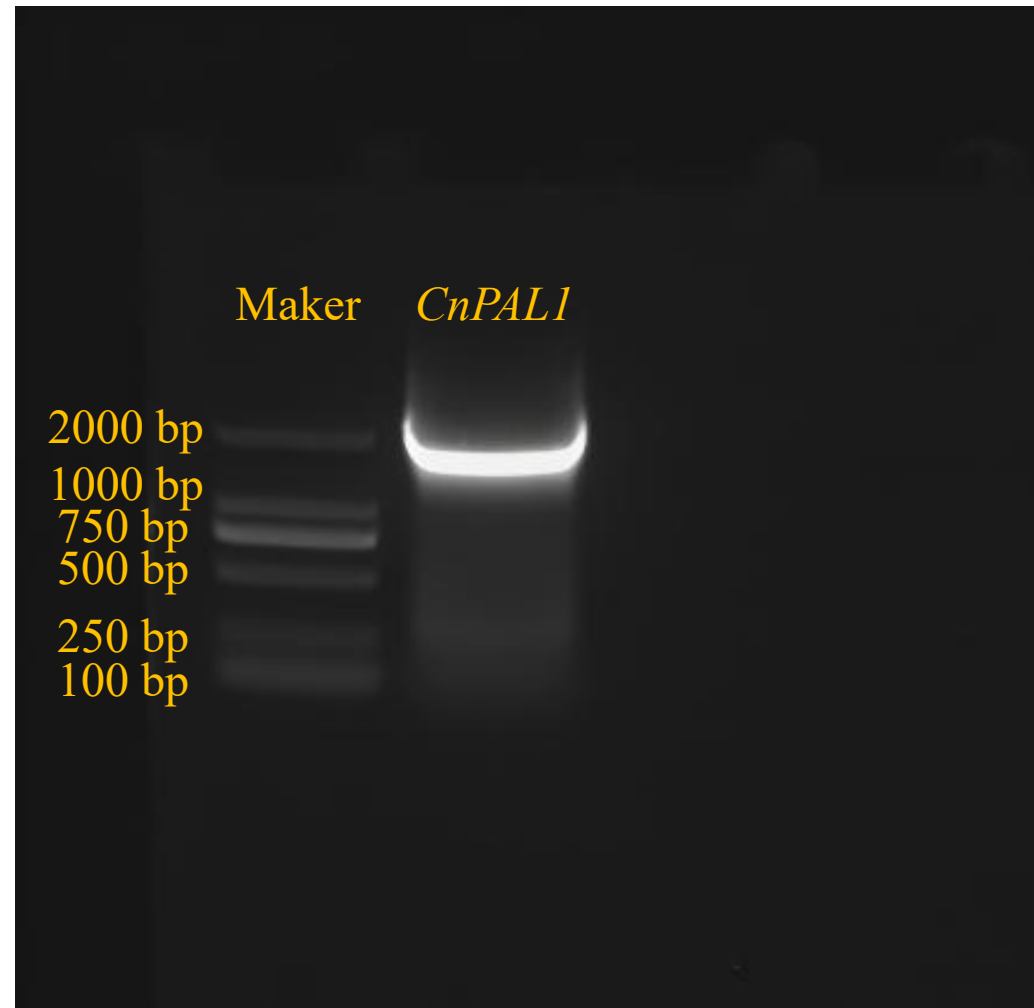

Supplement: Supplementary file 1 [file genes-16-01251-s001.zip › Figure S1 PCR amplification of CnPAL1 in Camellia nitidissima.pdf]

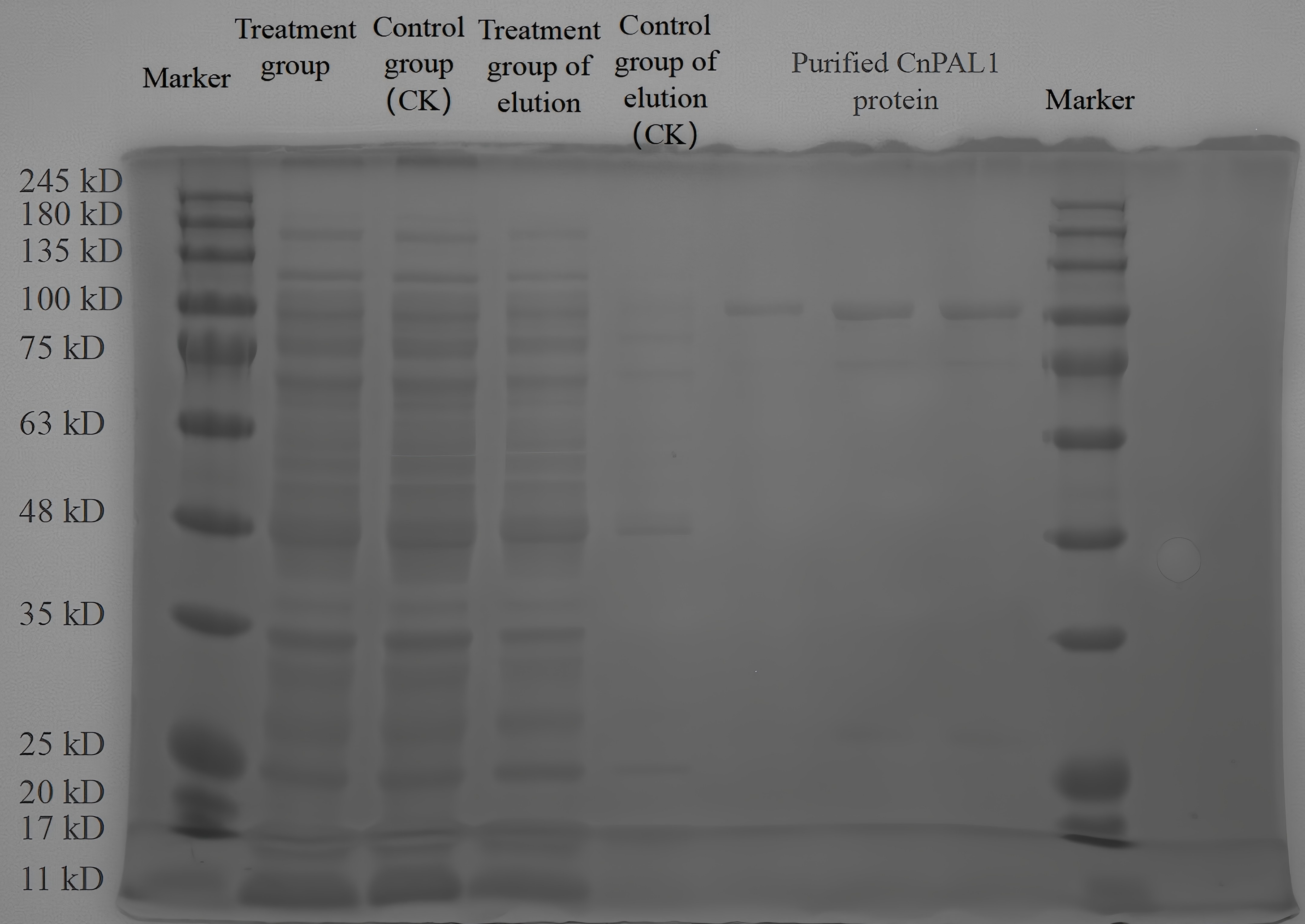

Supplement: Supplementary file 1 [file genes-16-01251-s001.zip › Figure S2 The expression and purification of CnPAL1 protein.pdf]

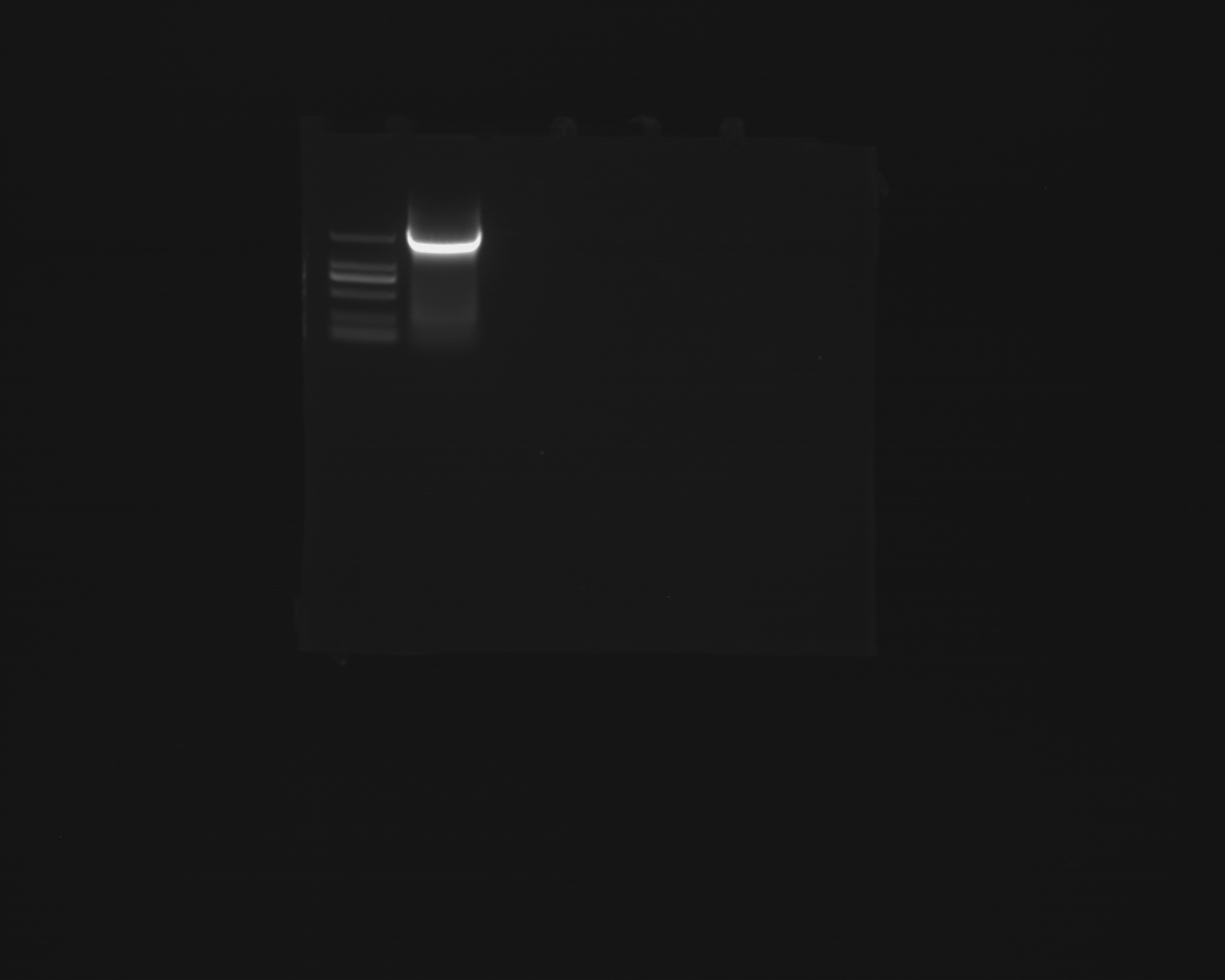

Supplement: Supplementary file 1 [file genes-16-01251-s001.zip › origin picture of Figure S1.tif]

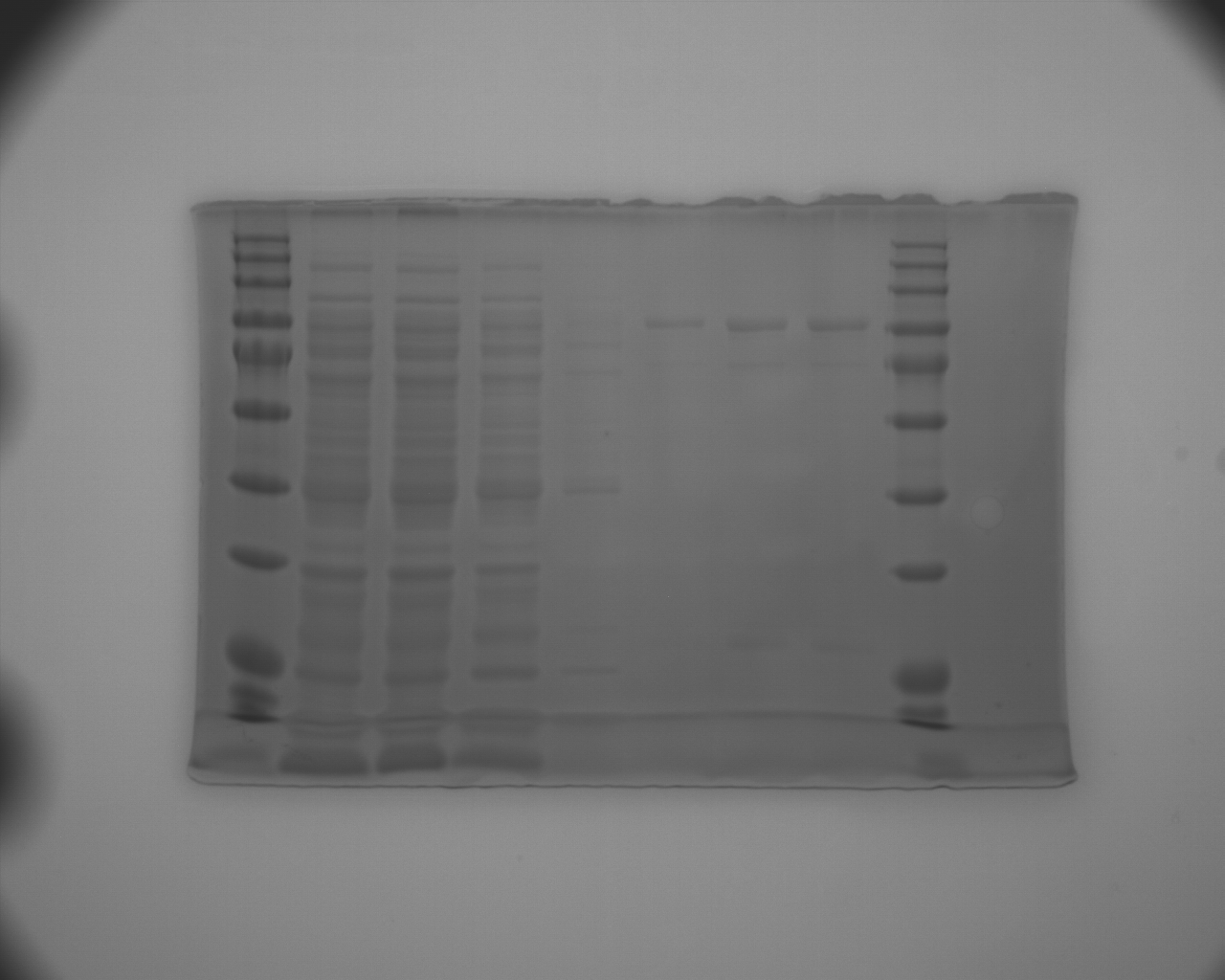

Supplement: Supplementary file 1 [file genes-16-01251-s001.zip › origin picture of Figure S2.tif]
